# Spectrum of myocardial involvement in patients with COVID-19 – An echocardiography study

**DOI:** 10.34172/jcvtr.31668

**Published:** 2024-03-13

**Authors:** Manoj kumar Dubey, Avinash Mani, Vineeta Ojha

**Affiliations:** ^1^Department of Medicine, SNMCH, Dhanbad, Jharkhand, India; ^2^Department of Cardiology, Fortis Escorts Heart Institute, New Delhi, India; ^3^Centre for Advanced Research in Imaging, Neuroscience and Genomics, Mahajan Imaging, New Delhi, India

**Keywords:** Covid-19, Global longitudinal strain, Myocarditis

## Abstract

**Introduction:**

Covid-19 patients can have both regional and global ventricular dysfunction. We aim to study the spectrum of myocardial involvement in Covid-19 patients on echocardiography.

**Methods:**

This is a single center, observational study where wall motion abnormality patterns were studied in Covid-19 patients along with global and regional longitudinal strain analysis (GLS).

**Results:**

30 Covid-19 patients were included in the study, with a mean age of 35.3±6.4 years. Echocardiography revealed characteristic wall motion abnormality involving hypokinesia of anterolateral and apical segments, which produced an operculum like appearance in all patients. Strain derived ejection fraction(EF) was lower in 4 chamber as compared to 2 chamber indicating regional myocardial dysfunction. Reduced GLS values in presence of normal EF indicates global systolic function impairment. Endocardial effacement was also noted in these segments along with stretching of interventricular septum.

**Conclusion:**

Specific myocardial involvement pattern can be detected on echocardiography, thus helping in diagnosis of Covid myocarditis.

## Introduction

 Cardiovascular involvement has been well documented in COVID-19 patients. The clinical presentation can include acute coronary syndrome, myocarditis, heart failure, ventricular arrhythmias and pericardial effusion.^1,2^ Myocarditis in Covid-19 patients is attributed to direct myocardial injury and immune mediated damage, with clinical presentation ranging from mild troponin elevation to decompensated heart failure.^3,4^ Cardiovascular magnetic resonance (CMR) imaging is considered as the gold standard for the diagnosis of myocarditis using the Lake Louis criteria.^5^ However, the lack of easy availability of CMR remains a major hurdle and highlights the importance of echocardiography in decision making.

 2D echocardiography in Covid-19 patients has revealed left ventricular dysfunction along with wall motion abnormalities.^6^ Right ventricular dysfunction is also noted in a quarter of patients. Patients with myocarditis can have a dilated ventricle with either global or segmental abnormalities.^7^ No specific myocardial involvement pattern has been identified in Covid-19 patients. Establishing causal relationship between myocardial involvement and Covid-19 may be difficult using echocardiographic alone. In the current study, we aimed to identify specific myocardial involvement patterns in Covid-19 patients. Identification of specific patterns can help establish echocardiography as a sensitive diagnostic modality for identifying Covid heart.

## Materials and Methods

 This was a prospective, observational study done at a tertiary care center, over a period of 6 months from January 2021 to June 2021. Covid-19 patients, diagnosed using positive RT-PCR and ECG changes were included in the study. Patients with severe Covid-19 and multisystem organ involvement were excluded from the study. All patients underwent transthoracic echocardiogram using Vivid T8 (GE Healthcare systems, Chicago, USA). Left and right ventricular function were assessed along with LV strain analysis(AFI). Post systolic strain index (PSI) was also calculated from strain analysis.

 All data was recorded in a tabular format and presented as summary statistics. Student’s t-test was used to estimate significant difference between ejection fractions measured in 4 chamber and 2 chamber. Pearson correlation analysis was done to determine association between longitudinal strain and PSI. A p value of < 0.05 was considered as significant.

## Results

 A total of 30 patients were included in this study. The mean age of the study population was 35.3 ± 6.4 years (range 14-50 years) and females comprised 45% of the study population. Fever and chest pain were the most common symptoms noted (35% and 30% respectively) (Table 1). All patients had elevated biomarkers in the study population which shows the prevalent pro-inflammatory state in these patients (Table 1). Half of the study group had rhythm abnormalities in the form of sinus tachycardia (n = 10), sinus bradycardia (n = 3) and atrial fibrillation (n = 2).

**Table 1:** Baseline demographics of Covid-19 patients

**Variables**	**N=30**
Age, years	35.3 ± 6.4
Females	14 (45)
Diabetes	0
Hypertension	0
Dyslipidemia	0
Smoker	0
Symptoms	
Asymptomatic	5(15)
Fever	11(35)
Chest pain	9(30)
Dyspnea	3(10)
Palpitations	2(6.6)
Cough	2(6.6)
Spo2, %	96.8 ± 0.9
Rhythm abnormalities	
Normal sinus rhythm	15(50)
Sinus tachycardia	10(33)
Sinus bradycardia	3(10)
Atrial fibrillation	2(7)
ST/T changes on ECG	12(40)
IL-6, pg/ml	11.4 ± 1.75
D-dimer, ng/ml	0.59 ± 0.14
Serum CRP, mg/L	10 ± 2.23
Serum Ferritin, ng/ml	1343.3 ± 33.6

Age, Spo2 and troponins are presented as mean ± SD whereas time duration post Covid-19 is presented as median. Rest all values are expressed in percentages. Spo2 – Room air saturation.

**Figure 1:**
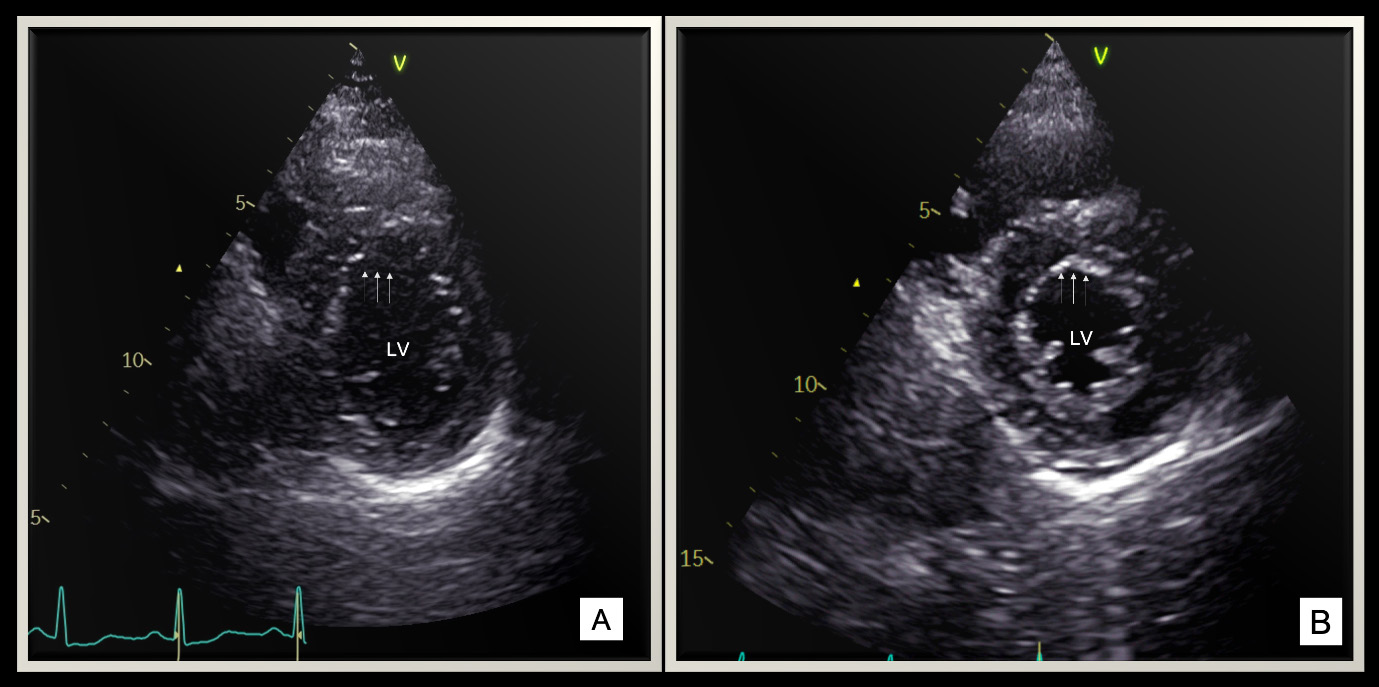
(A). Short axis view(SAX) at mid ventricular level showing hypokinesia of anterior and anterolateral segment (denoted by blue arrows). (B). The characteristic wall motion abnormality produces an appearance of an operculum resting on top of the heart

 Echocardiography revealed normal sized left ventricle and preserved systolic function with a mean M-mode ejection fraction (EF) of 58.3 ± 6.7% in the study group. However, strain derived auto EF was significantly lower in 4 chamber (4C) as compared to 2 chamber(2C) (58.7 ± 4.7% vs 55.5 ± 4.1%, *P* = 0.002). This points towards the presence of regional myocardial dysfunction in these patients. All patients had myocardial involvement in the form of hypokinesia predominantly involving anterolateral, apicoseptal and apicolateral segments as compared to other segments (Figure 1 A, Table 2).

**Table 2:** Echocardiographic characteristics of patients with Covid-19

**Variables**	**N=30**
LV internal dimension diastole, mm	44 ± 3.7
LV internal dimension systole, mm	30.3 ± 3.6
LV ejection fraction (M-mode), %	58.3 ± 6.7
LV ejection fraction (4 CH), %	55.5 ± 4.1
LV ejection fraction (2CH), %	58.7 ± 4.7
Wall thickness, mm	8.7 ± 1.1
Wall motion abnormality, %	
Anteroseptal	14(45)
Anterior (basal)	16(53)
Anterolateral	30(100)
Inferoseptal	2(6.6)
Inferior (Basal)	0
Inferolateral	0
Anterior (apex)	30(100)
Septal (apex)	30 (100)
Inferior (apex)	0
Lateral (apex)	30(100)
RV systolic pressure, mmHg	14.8 ± 3.9
TAPSE, mm	18.5 ± 2.1
RV FAC, %	46.2 ± 4.4
Diastolic dysfunction, %	6 (20)
Intracardiac thrombus, %	0

LV dimensions, wall thickness, longitudinal strain, PSI, peak systolic delay and RV systolic pressure are denoted as mean ± SD. Rest all variables are expressed as percentages. RV FAC – RV fractional area change, TAPSE – Tricuspid annular plane systolic excursion.

 This characteristics wall motion abnormality produces an “operculum” like appearance over the other segments in the short axis view in systole (Figure 1B, Supplementary Video 1). Endothelial involvement was characterized by predominant involvement of interventricular septum (IVS) producing a characteristic jerky motion and stretching of IVS.

 Global longitudinal strain (GLS) was reduced in 45% of the study population (Table 3). The anterior and apicoseptal segments had maximum reduction in longitudinal strain values amongst all patients. Peak systolic index was elevated in the involved segments indicating contractile abnormalities. A high degree of correlation was seen between regional longitudinal strain and segmental PSI, demonstrating impaired systolic contraction in the affected segments (r = 0.702, *P* = 0.001). No gender difference in GLS values were noted in the study population (*P* = 0.237).

**Table 3:** Global longitudinal strain and PSI assessment in the study population

**Variables**	**N=30**
Mean global longitudinal strain (GLS)	- 17.2 ± 1.97
Patients with reduced GLS	14 (45)
Average number of segments with reduced longitudinal strain	3.4 ± 1.8
Mean LS in affected segments	-10.5 ± 2.3
Segments with reduced longitudinal strain, %	
Anteroseptal	12 (40)
Anterolateral	18 (60)
Inferoseptal	0
Inferolateral	0
Anterior	27 (90)
Inferior	0
Septal	2 (6.6)
Lateral	21 (70)
Number of segments with increased PSI	2.3 ± 1.2
Segments with increased PSI, %	
Anteroseptal	5 (15)
Anterolateral	21 (70)
Inferoseptal	0
Inferolateral	0
Anterior	24 (80)
Inferior	0
Septal	0
Lateral	0

GLS – global longitudinal strain, PSI – peak systolic strain index, PSD – peak systolic delay.

## Discussion

 The current study intended to evaluate the spectrum of myocardial involvement in Covid-19 patients using echocardiography. Echocardiography revealed reduced global ventricular function as demonstrated by reduced GLS in about half of the study population. All patients had a characteristic wall motion abnormality producing an operculum like appearance in systolic frames. There was predominant involvement of anterior and apical segments as compared to other segments leading to reduced strain derived 4C ejection fraction. Endothelial involvement was noted along with elevated PSI values. To our knowledge, this is the first study which aims to study myocardial involvement pattern noted in Covid-19 patients.

 Echocardiography is an invaluable and easily accessible tool for diagnosis of Covid-19 associated myocarditis. Echocardiographic findings in Covid-19 patients were evaluated in a large study conducted by Dweck et al.^6^ Left ventricular abnormalities were noted in 39% patients whereas myocarditis was seen in 3%. In our cohort, patients had global ventricular dysfunction as evidenced by reduced GLS as well as regional predilection as evidenced by characteristic motion abnormalities noted in anterolateral and apical segments. Regional involvement in Covid-19 patients has been documented, ranging from basal and mid ventricular involvement to limited hypokinesia of inferior/inferoseptal segment.^8^ A systematic review of cardiac MRI in 199 Covid-19 patients revealed that global hypokinesia was the most common myocardial involvement pattern in these patients.^9^ On the contrary, our study patients had hypokinesia involving anterolateral and apical segments, giving the appearance of an operculum. This was coupled with effacement of the endocardial lining in these segments and stretching of IVS. This characteristic pattern noted in our patients is a unique one, which has not been described before in Covid-19 patients.

 Global longitudinal strain is a sensitive marker for myocardial dysfunction. Echocardiographic evaluation of hospitalized Covid-19 patients revealed that global longitudinal strain was reduced in patients with preserved LV ejection fraction, as noted in our patients.^10^ PSI analysis is also a surrogate marker for evaluation of ventricular dysfunction. Longitudinal strain values were reduced in the involved segments whereas PSI values were elevated, signifying regional myocardial dysfunction. Covid-19 infection has been postulated to cause coronary microvascular dysfunction (CMD) which can lead to subendocardial ischemia. Subendocardial perfusion defects have been demonstrated in patients with history of Covid-19 infection using cardiac MRI.^11^ CMD could be the probable cause for wall motion abnormalities and endocardial effacement noted in our patients. Coronary microvascular dysfunction may cause global wall motion abnormalities which slowly recovers over time. The rate of recovery can be variable for individual segments, with anterior and apical segments showing slower recovery as compared to others, thus producing the characteristic appearance on echocardiogram. Newer evidence pertaining to disturbance of regional perfusion instead of global perfusion, post severe Covid-19 infection is emerging.^12^ Follow up studies need to be undertaken to determine the long-term impact of Covid-19 on myocardium, regarding the reversibility of initial changes.

 The current study has its inherent limitations. The small sample size prohibits us from generalizing the results to the entire spectrum of Covid-19 patients. CMR imaging could not be performed in these patients due to lack of availability and financial constraints. As this was a single center hospital-based study, selection bias could have influenced the results, with sicker patients being excluded from the analysis.

## Conclusion

 Covid-19 patients have global as well as regional myocardial dysfunction. Specific echocardiographic pattern can help identify myocardial involvement in Covid-19 patients. Echocardiography should be considered the modality of choice in such situations.

## Competing Interests

 None of the authors have any conflict of interests

## Ethical Approval

 Ethics approval was obtained from the Institutional ethics committee (IEC/2021-167) and informed consent obtained from the participants.

## Funding

 None.

## Supplementary Files

Supplementary video 1. Echocardiographic clip of short axis view at mid ventricular level shows hypokinesia of anterior and anterolateral segments, producing an operculum like appearance. Rest all segments are contracting normally.
